# The illusion of a “sense of body lightness” while walking: a preliminary exploratory study

**DOI:** 10.3389/fpsyg.2026.1741215

**Published:** 2026-02-19

**Authors:** Kazuki Hayashida, Yuki Nishi, Kazuki Osawa, Yasuhiro Inui, Shu Morioka

**Affiliations:** 1 Department of Rehabilitation, Faculty of Wakayama Health Care Sciences, Takarazuka University of Medical and Health Care, Wakayama, Japan; 2 Neurorehabilitation Research Center, Kio University, Umaminaka, Koryo, Kitakatsuragi-gun, Nara, Japan; 3 Institute of Biomedical Sciences (Health Sciences), Nagasaki University, Nagasaki, Japan; 4 Department of Rehabilitation, Kyoto Tanabe Central Hospital, Kyoto, Japan; 5 Department of Neurorehabilitation, Graduate School of Health Sciences, Kio University, Umaminaka, Koryo, Kitakatsuragi-gun, Nara, Japan; 6 Department of Rehabilitation, Nara Prefecture General Rehabilitation Center, Nara, Japan

**Keywords:** illusion of sense of body lightness, sense of agency, sense of ownership, sensory-motor incongruence, visual delay feedback

## Abstract

Illusions have historically been used in experimental psychology to reveal information about perceptual processes. A recent study reported that participants felt a sense of body heaviness when given slightly delayed (incongruent) visual feedback compared with the predicted somatosensory feedback. In this study, we reported a novel illusion of feeling a sense of body lightness while walking. There is consensus that an important factor in a body’s perceptual process is congruency between the senses. When our 30 participants experienced “subjectively preceding feedback” while walking on a treadmill, nine of them felt a sense of body lightness. In this report, we discussed how we were able to generate the subjectively preceding feedback, the mechanism that induces the illusion of a sense of body lightness, and the potential applications of this illusion. Although this study was just a preliminary and exploratory research, this new illusion has the potential to contribute not only to the medical and rehabilitation fields but also to extended reality technology and other interdisciplinary fields.

## 1 Introduction

Since the rubber hand illusion was first reported (Botvinick and Cohen, 1998), there has been consensus that an important factor in embodiment sensations (sense of ownership and sense of agency) is congruency between the senses or between the prediction and the feedback. Sense of ownership refers to the experience of mineness toward a body part, while sense of agency is the experience of motor control over one’s body actions. Today, this knowledge has been applied in cognitive psychology (Ehrsson et al., 2004; Tsakiris and Haggard, 2005; Ehrsson, 2007; Lopez et al., 2008; Tsakiris, 2010; Seghezzi et al., 2019; Arzy and Schacter, 2019), the medical and rehabilitation fields (Burin et al., 2015; Tieri et al., 2018) and living environments, including smartphones (Cattan et al., 2018). In the motor control theory of the feedforward model, when motor intention occurs, predicted sensory feedback is generated in the brain and integrated with actual sensory feedback (Wolpert and Ghahramani, 2000; Blakemore et al., 1998; Blakemore et al., 1999; Frith et al., 2000). When the predicted sensory and actual sensory feedback are congruent, a sense of agency occurs, but when they are incongruent, uncomfortable movement and negative feelings occur. For example, patients with musculoskeletal and central nervous system disorders experience feedback of motor trajectories and sensations that differ from the predicted outcomes with motor intention. This incongruence reduces self-attribution and often results in negative sensations such as heaviness, discomfort, and pain associated with movement (McCabe et al., 2005; Foell et al., 2013; Kuppuswamy et al., 2016). These patients experience a negative sensation of incongruency every time they move, and even moving itself becomes an uncomfortable experience.

One experimental method often used to investigate the mechanisms of feeling generation induced by incongruence is to manipulate the spatial mismatch between the senses (Katayama et al., 2016; Katayama et al., 2019). In this experiment, when spatial incongruence is induced between visual feedback of one upper extremity and somatosensory feedback of another upper extremity using a mirror, a negative sensation occurs. Other experiments use a method for manipulating temporal incongruence. For example, it is known that the temporal perception of an intention-based voluntary action and the feedback induced by the action are attracted to each other (action and feedback); this phenomenon is called intentional binding. Intentional binding is recognized as a method that can be interpreted as sense of agency decreasing as the perceived time delay between an intention-based action and its feedback increases (Haggard et al., 2002; Moore and Obhi, 2012; Haggard, 2017).

Another typical method for manipulating sensorimotor incongruence has been studied using visual feedback delay (Shimada et al., 2009; Shimada et al., 2010; Shimada et al., 2014). In these studies, various temporal incongruities between the actual movement and visual feedback were introduced using a visual feedback delay experimental setup. The delay time was systematically varied, and the participants responded to whether the predicted and actual feedback were congruent or incongruent. Studies have shown that the rate of responses indicating congruence decreases when the delay increases. A study using wrist motion also reported that body perception (sense of body heaviness) and motor data (electromyography data) can be disturbed by manipulating the incongruence with a time delay (Osumi et al., 2018). Sensory integration through this temporal congruence has also been helpful in the sense of ownership (Shimada et al., 2009).

Although these experiments induced an incongruity focused on upper-extremity movement, Kannape and Blanke (2013) reported an interesting experiment on sensory-motor incongruence of whole-body movement while walking using visually delayed feedback in healthy participants. Participants were shown delayed visual feedback while walking on a treadmill, which was instantly converted into an avatar using 3D motion analysis equipment. Similar to a recent study involving the upper extremities, the rate of responses indicating congruence decreased when the delay time increased. With reference to this recent study on the temporal incongruency method while walking, we found that the illusion of a sense of body heaviness was induced (Hayashida et al., 2025). We believe that the incongruence of delayed visual feedback over the predicted sensory feedback induces the illusion of a sense of body heaviness. Therefore, these recent studies (Osumi et al., 2018; Hayashida et al., 2025) indicated that decreasing the sense of agency can indirectly induce the sense of body heaviness.

Interestingly, in the recent study (Hayashida et al., 2025), some of our participants reported a positive sensation of an illusion of body lightness during the experience of incongruence. Why was this illusion induced? The easiest and most easily imaginable example of a light feeling in the body is the sensation of walking on the moon. If 1/6 of Earth’s gravity is fed back on the Moon, as opposed to the predicted gravity on Earth, the body should feel lighter for that amount of gravity. Let us reiterate that the illusion of a sense of body heaviness is induced by an incongruence between delayed and predicted sensory feedback. In contrast, here we propose that the illusion of a sense of body lightness might be induced by incongruence between “subjectively preceding feedback” and predicted sensory feedback.

**Figure 1:**
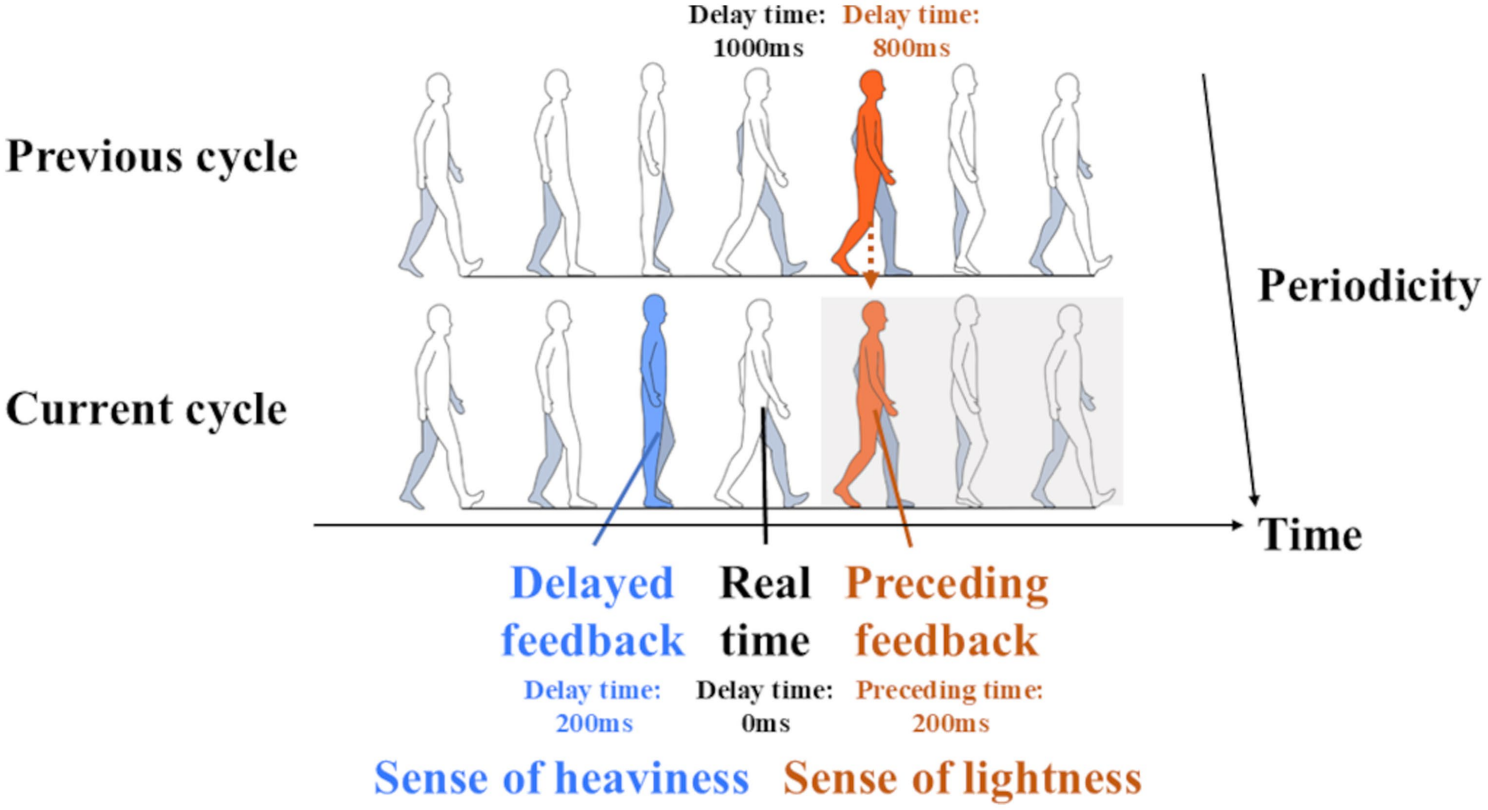
Subjectively preceding feedback method. In this situation, the participant has a single stride time of 1,000 ms, and the real time is the right “terminal stance phase.” On the one hand, when the participants were given visual feedback through the camera as a “mid-stance phase” (current cycle blue; delay time 200 ms), they experienced a sense of body heaviness. They had the sensation of watching themselves a little into the past. On the other hand, when participants were given feedback in the right “pre-swing phase” situation (previous cycle orange) with a delay of nearly one stride (delay time: 800 ms), they had the sensation of watching themselves a little into the future and not the past (current cycle orange; preceding time: 200 ms). Each value used here is merely an example.

Because walking is a periodic movement, introducing a delay close to one stride can result in visual feedback that precedes the ongoing movement. As shown in Figure 1, when a participant’s single stride time is approximately 1,000 ms, real-time feedback (delay time: 0 ms) corresponds to the terminal stance phase. In contrast, when feedback is presented during the pre-swing phase with a delay of nearly one stride (e.g., 800 ms; Figure 1, previous cycle, orange), the visual information corresponds to a slightly future phase of the gait cycle (preceding time: 200 ms; Figure 1, current cycle, orange). This configuration effectively provides what we term “subjectively preceding feedback.” Such predicted feedback could induce an illusory sense of body lightness in some participants, and the purpose of this study was to investigate this systematically.

In addition, the fact that the illusion of a sense of body lightness was induced in only some participants may have been owing to psychological characteristics. In particular, sense of agency, the subjective sensation produced by the integration of predicted and actual sensations, is affected by psychological traits. Schizophrenic personality and depression have been reported as the psychodynamic factors associated with a sense of agency. Higher schizophrenic personality tendencies are associated with reduced sensory-motor processing, which is thought to alter subjective experiences (Moore et al., 2011). It has also been reported that those with higher depressive tendencies are less able to incorporate feedback well (Obhi et al., 2013; Malik and Obhi, 2019; Nobusako et al., 2020) and have a reduced sense of agency. Therefore, it may be important for the illusion of lightness to induce a sense of agency for the visual feedback on the monitor; that is, it may be necessary to have a psychological feature that may allow immersion in it. In addition, individuals with psychological traits that are sensitive to sensory feedback may have superior sensory-motor processing, which may impact the sensory-motor integration process. This psychological trait, including sensory sensitivity, may be related to the acceptability of sensory feedback and may have influenced the elicitation of a sense of body lightness.

In summary, incongruence between predicted and actual feedback in upper limb movements causes negative effects, such as a sense of body heaviness. However, it is theoretically possible to induce positive sensations of a different nature than sense of body heaviness, such as a sense of body lightness, by using visual delay tasks while walking. Many patients with neurological and musculoskeletal disorders suffer from body perception disturbances, including a sense of body heaviness (Kuppuswamy et al., 2016; Serrada et al., 2021; Matsuda and Osumi, 2023; Dagenais et al., 2025). If it is possible to intentionally induce a sense of lightness in contrast to the negative sensation of body heaviness that many patients experience, this may be used as a future intervention tool. This new illusion has the potential to contribute not only to the medical and rehabilitation fields but also to extended reality technology and other interdisciplinary fields. Therefore, the purpose of this study was to investigate whether the application of a visual delay system while walking could induce an illusion of a sense of body lightness, and to investigate the relationship between this illusion and psychological characteristics.

This study was exploratory and preliminary in nature. Accordingly, the exploratory questions were as follows.

*RQ1*: We examined whether a visual delay system during walking induces a sense of bodily lightness and determined its prevalence. We attempted to classify participants into an Illusion group and a Non-illusion group based on the definition described below.

*RQ2*: Previous studies (Osumi et al., 2018; Hayashida et al., 2025) have shown that participants recognize sensorimotor incongruence when experiencing a sense of body heaviness. However, it remains unclear whether participants recognize sensorimotor incongruence when experiencing a sense of body lightness. If some participants could be classified into the Illusion group, we investigated the congruence response rate under the minimum sensorimotor incongruence condition, the body heaviness condition, and the body lightness condition.

*RQ3*: We investigated differences in the congruence response rate between groups under the minimum sensorimotor incongruence condition and the body heaviness condition, because, at a more fundamental level, groups may differ in their attitudes toward sensorimotor incongruence.

*RQ4*: We compared psychological traits between groups, as these traits may be related to the acceptability of sensory feedback and may influence the elicitation of a sense of body lightness.

## 2 Methods

### 2.1 Participants

It was difficult to determine the sample size of this study *a priori* because it was quite novel and the proportion of participants who felt a sense of body lightness was completely unknown. We used our study (Hayashida et al., 2025) as a rough reference to determine the sample size. Thirty healthy students (15 females; mean age = 21.8, standard deviation = 0.8) participated in the experiment. A *post hoc* power analysis was performed to validate the sample size (see below). All participants reported normal functioning required for the experiment. The Kio University ethics committee approved the study procedures (R3-41), and the researchers conducted the experiment in accordance with the Declaration of Helsinki. The study, including participant information, setup, and breaks, lasted for 70 min.

### 2.2 Devices and software

MATLAB R2022a (MathWorks Inc., Natick, MA, United States) was used to design the paradigm, and a 27-inch monitor (165 Hz) was used as the presentation screen (VG278QR-J, ASUS, Tokyo, Japan). A personal computer (FUJITSU LIFEBOOK UH90/C3, Minato City, Japan) was used for all tasks, and to record the data.

### 2.3 Procedure

The participants walked continuously on a treadmill (SplitR; SENSTYLE, Japan) for an entire experimental block. Participants were instructed to swing their arms as they do during overground walking and only use the handlebar if they lost balance. Participants received visual feedback (800 × 600 pixels) on their movements using the monitor. The distance between the participant and the projection screen was 185 cm.

### 2.4 Paradigm

**Figure 2:**
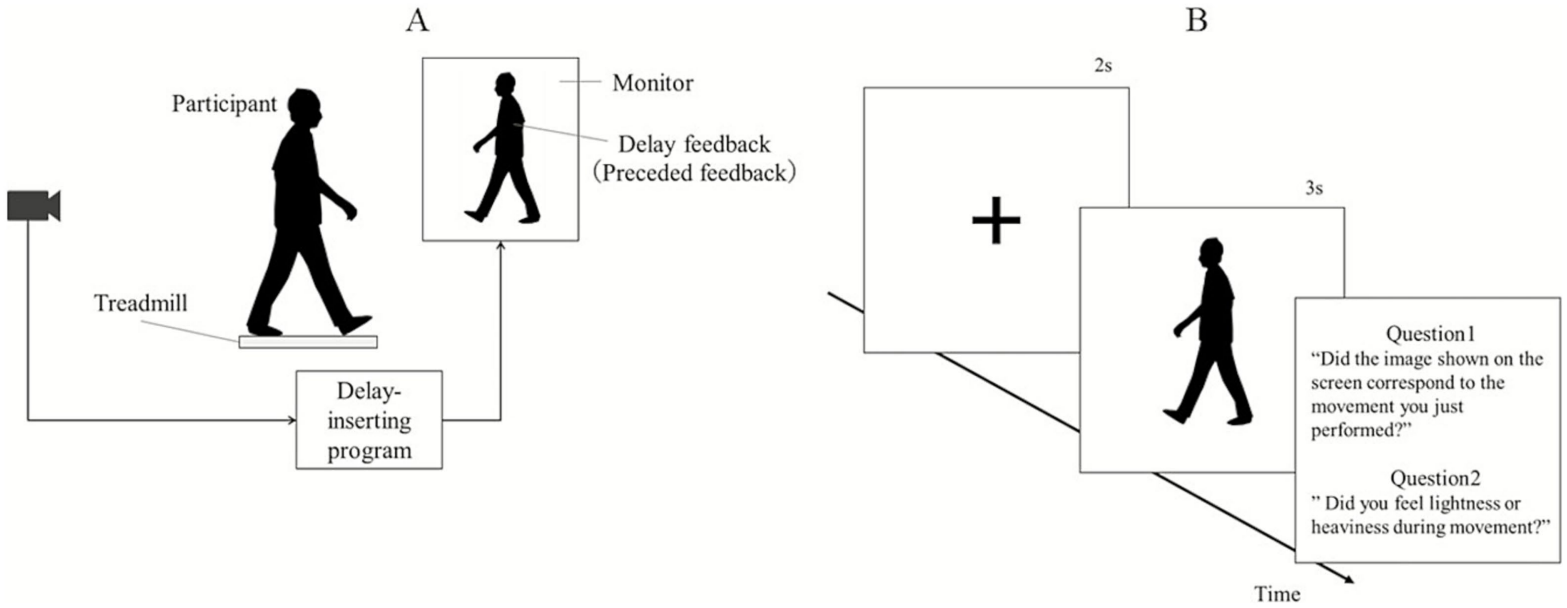
Experimental procedure. **(A)** Participants walking on a treadmill watched visual feedback of themselves taken from behind by a camera with a randomized additional delay. This represented a time delay of half of one stride time visual feedback: when the participant’s left leg was backward, the body shown on the monitor was positioned with the right leg backward. If the visual feedback was presented in real-time (i.e., minimum delay), when the participant’s left leg was backward, the body shown on the monitor was similarly positioned with the left leg backward. **(B)** Each trial began with only the fixation cross shown on the screen (2 s). Subsequently, participants received visual feedback of their walking for 3 s, after which they were asked via a pop-up text box: question 1 (Q1) “Did the image shown on the screen correspond to the movement you just performed?” (congruent-incongruent detection) and question 2 (Q2) “Did you feel lightness or heaviness during movement?” (sense of body lightness or heaviness). Participants responded orally with Yes/No for Q1 and using a 7-point Likert scale for Q2 (1, very light, 4, no feeling, 7, very heavy).

First, the participants were given time to familiarize themselves with the treadmill, mostly within 5 min. The treadmill speed was maintained at 1 m/s throughout the experiment. In each trial, the participants watched visual feedback of themselves taken from behind by a camera with a randomized additional delay (170, 195, 220, 245, 270, 320, 370, 470, 570, 820, 920, 1,020, 1,120, 1,220, and 1,320 ms; 8 trials per delay). These conditions included an intrinsic delay (170 ms) for the entire system. The intrinsic setup delay varied within 11 ms. A schematic of the setup is shown in Figure 2. Each trial began with only a fixation cross displayed on the screen (2 s). Subsequently, participants received visual feedback of their walking for 3 s, after which they were asked via a pop-up text box: question 1 (Q1) “Did the image shown on the screen correspond to the movement you just performed?” (congruent-incongruent detection, Franck et al., 2001; Kannape and Blanke, 2013) and question 2 (Q2) “Did you feel lightness or heaviness during movement?” (sense of body lightness or heaviness; Osumi et al., 2018). Participants responded orally with Yes/No for Q1 and using a 7-point Likert scale for Q2 (1: very light, 4: no feeling, 7: very heavy), which was recorded by the experimenter. Participants reported posteriorly at the end of all the trials whether they had experienced the subjectively preceding feedback when a sense of body lightness was induced. A practice phase of at most five trials was conducted after ensuring that the participants fully understood Q1 and Q2. During the practice phase, all participants were not able to notice whether the feedback was real-time (i.e., minimum delay) or delayed by one full cycle. The participants wore closed headphones (Soundcore, Life Q35Anker, United States) that played white noise and used noise cancellation to remove auditory feedback from their footfalls. Participants walked continuously throughout all trials, including the response phase at the end of each trial. All participants wore patternless, plain clothing fitted to their body shapes for the experiment. Participants were not able to adapt to the delayed feedback, as feedback only lasted for 3 s, 1 or 2 strides on average, and delays were randomized across trials.

### 2.5 Psychological assessment

Regarding the Beck Depression Inventory (BDI)-II, higher scores indicate more severe depressive states (Beck et al., 1996; Nishiyama and Sakai, 2009). Furthermore, regarding the Schizotypal Personality Questionnaire (SPQ), higher scores indicate more severe schizotypy states (Raine, 1991; Iijima et al., 2010). To measure the severity of the sensory sensitivity states, we used the Highly Sensitive Person Scale (HSPS-J19), with higher scores indicating more severe sensory sensitivity (Aron and Aron, 1997; Takahashi, 2016).

### 2.6 Statistical analyses

**Figure 3:**
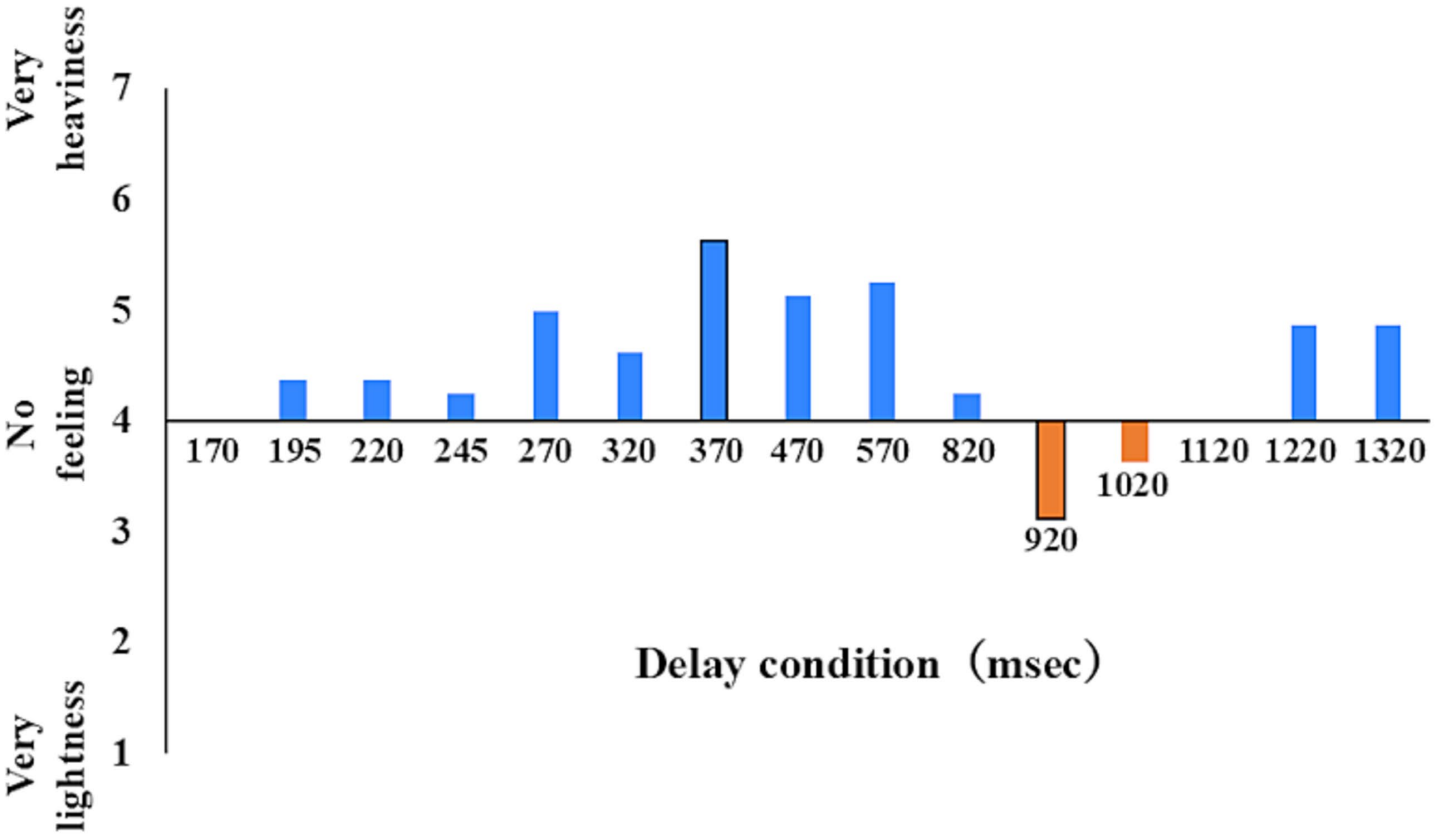
Body perception at each delay level; examples of personal data. This value is the mean of each delay time for one participant of the illusion group in Q2. We defined the illusion group based on the mean score of the sense of body (Q2) being less than 4 in at least one delay condition. Next, in the illusion group, we extracted the rate of response of the congruence of each of the three delay conditions: the no incongruence condition (here 170 ms, minimum delay condition), the highest sense of body heaviness delay condition (here 370 ms, heaviness condition), and the highest sense of body lightness delay condition (here, 920 ms, lightness condition).

RQ1: We divided the participants into two groups: illusion of a sense of body lightness (Illusion) and Non-illusion groups. We defined the Illusion group as participants whose mean Q2 score was below 4 in at least one delay condition (Figure 3) and who reported Q2 scores below 4 in at least four out of eight trials within that condition. RQ2: In the Illusion group, we extracted the congruence response rate of each of the three delay conditions: the no incongruence condition (i.e., 170 ms condition, Minimum delay condition), the highest sense of body heaviness delay condition (370 ms in the example of Figure 3, Heaviness condition), and the highest sense of body lightness delay condition (920 ms in the example of Figure 3, Lightness condition), and analyzed these using a one-way ANOVA. The Bonferroni method was used for multiple comparisons. RQ3: We analyzed the congruence response rate of the Minimum delay condition and the Heaviness condition between groups using a two-way ANOVA.

As the responses to the lightness/heaviness question may reflect demand characteristics, we added the analysis to address this concern. If the participants in the Illusion group responded the high sense of body lightness due to having demand characteristic, those showing stronger sense of body lightness responses could respond also a higher sense of body heaviness. Based on the hypothesis, we analyzed the Spearman’s correlation between the minimum Q2 mean (i.e., sense of body lightness) and the maximum Q2 mean (i.e., sense of body heaviness) in each group. This analysis was conducted using Supplementary data.

RQ4: To investigate the characteristics associated with experiencing the illusion of body lightness, each psychological assessment measure was compared between groups. We analyzed each psychological assessment measure using the Mann–Whitney U test between groups. The Shapiro–Wilk test was used to test normality. *p* < 0.05 were considered statistically significant. SPSS Statistics for Windows, ver. 28 (IBM, Japan) was used for the statistical analysis.

## 3 Results

All participants completed the experimental task without missing their balance and responded to two questions within the 3 s.

**Figure 4:**
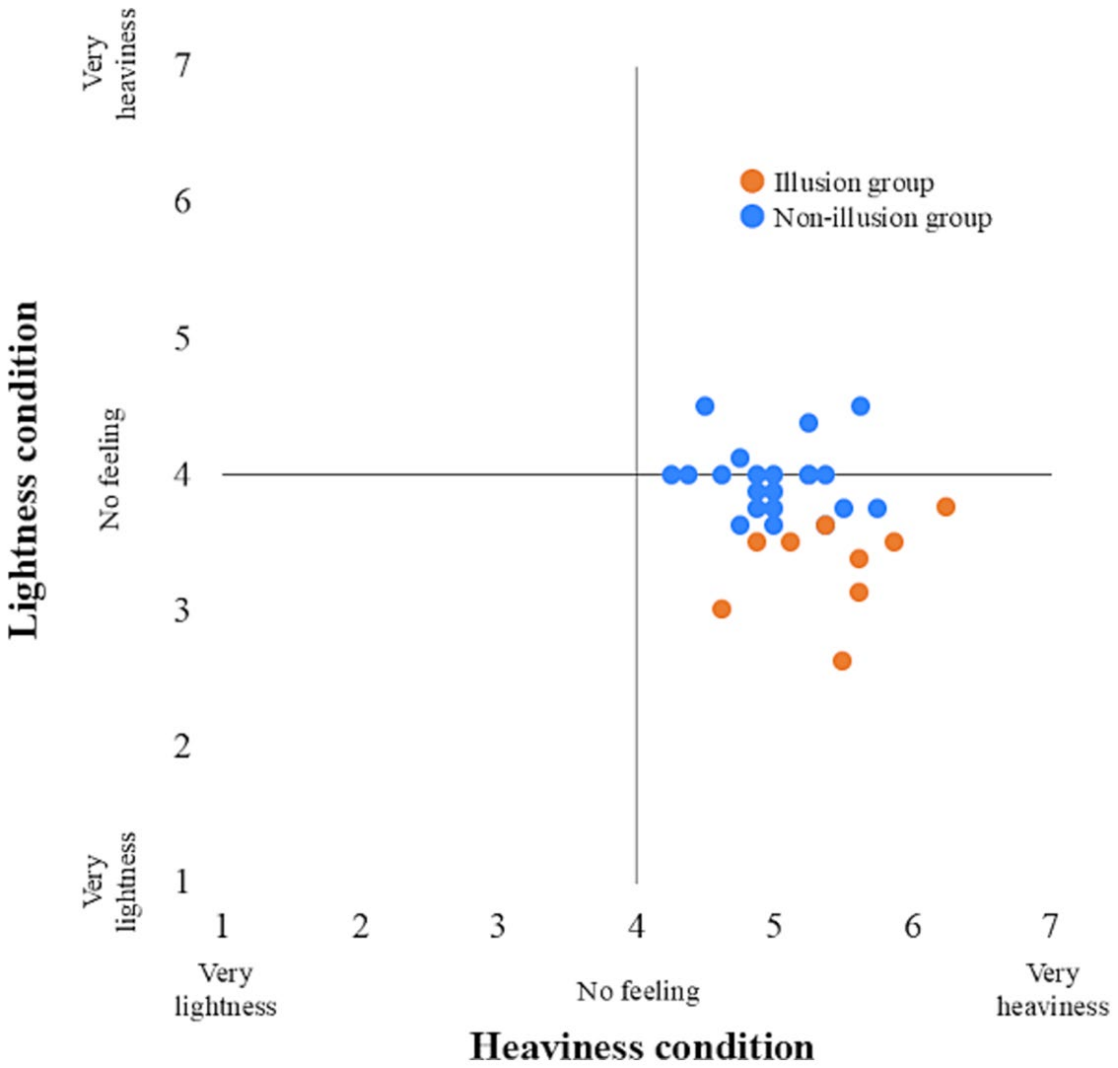
Individual plots across body perception between conditions. Orange represents the illusion group, and blue represents the non-illusion group.

**Figure 5:**
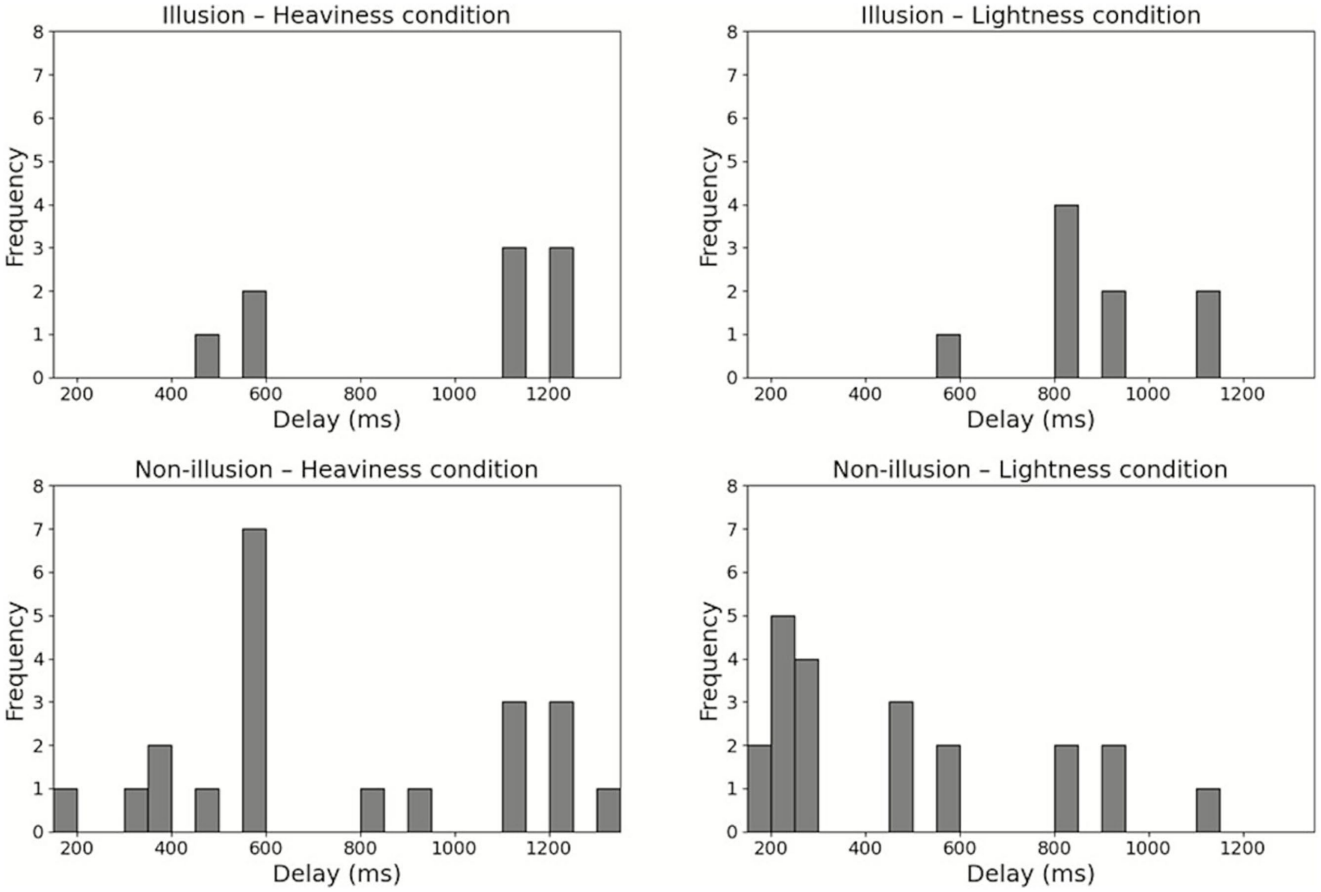
Histograms of each body perception and each delay condition. Distribution of delay conditions for each body perception in each group.

The participants were divided into an Illusion group (*n* = 9) and a Non-illusion group (n = 21). All participants in the Illusion group reported that they experienced the subjectively preceding feedback when they responded to the sense of body lightness. The minimum Q2 mean values (i.e., sense of body lightness) and the maximum Q2 mean values (i.e., sense of body heaviness) for each participant used in this grouping are shown in Figure 4. In the Illusion group, the median delay was 1,120 ms (interquartile range (IQR) = 570–1,220) in the Heaviness condition and 820 ms (IQR = 820–1,020) in the Lightness condition. In the Non-illusion group, the median delay was 570 ms (IQR = 520–1,120) in the Heaviness condition and 270 ms (IQR = 233–695) in the Lightness condition. The histograms of each body perception and each delay condition are shown in Figure 5.

**Figure 6:**
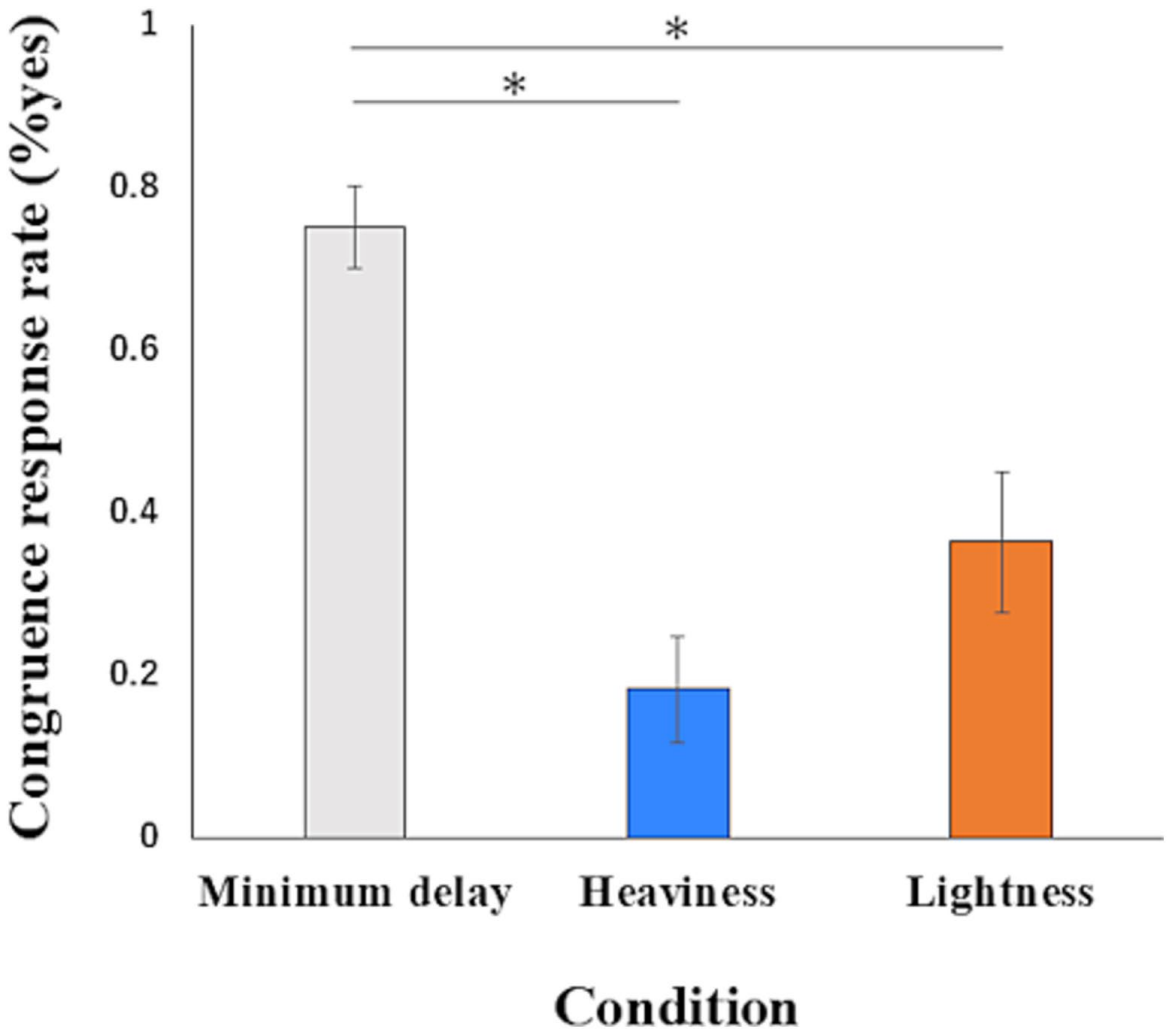
The data of the congruence response rate. Data represent means ± standard error. ∗*p* < 0.01.

The one-way ANOVA of the congruence response rate showed a significant main effect (*F*(2, 16) = 21.051, *p* < 0.01, η^2^_p_ = 0.725). A *post hoc* power analysis using G*Power 3.1.9.2 (Faul et al., 2007) indicated that the achieved power was high (Power ≈ 1.00). Multiple comparisons revealed that the congruence response rate in the Minimum delay condition (mean = 0.752, SD = 0.153) was significantly higher than in the Heaviness (mean = 0.182, SD = 0.198) (mean difference = 0.570, 95%CI = 0.413 to 0.727, p < 0.01) and Lightness conditions (mean = 0.364, SD = 0.261) (mean difference = 0.388, 95%CI = 0.118 to 0.658, p < 0.01). The congruence response rate in the Lightness condition and the Heaviness condition was not significantly (mean difference = 0.182, 95%CI = − 0.167 to 0.532, *p* = 0.46) (Figure 6). The two-way ANOVA of the congruence response rate showed no interaction (*F*(1, 28) = 1.562, *p* = 0.222, η2p = 0.053), no main effect in the group (*F*(1, 28) = 0.829, *p* = 0.370, η2p = 0.029) and main effect in the conditions (*F*(1, 28) = 224.261, p < 0.01, η2p = 0.889). In the main effect in the conditions, a *post hoc* power analysis using G*Power 3.1.9.2 (Faul et al., 2007) indicated that the achieved power was high (Power ≈ 1.00). The descriptive statistics of congruence response for each condition in the Non-illusion group were as follows: the Minimum delay condition (mean = 0.752, SD = 0.198) and the Heaviness condition (mean = 0.079, SD = 0.152).

The results of the correlation analysis between the minimum and maximum Q2 mean values show that no significant correlations were found in the Illusion group (*ρ* = 0.315, *p* = 0.409) or in the Non-illusion group (ρ = −0.168, *p* = 0.466). These results indicate that individuals who reported higher sense of body lightness did not necessarily report a higher sense of body heaviness, indirectly suggesting that the illusion group was not a population with high demand characteristics.

For the SPQ, there was no significant difference (*U* = 66.000, *r* = −0.285, *p* = 0.125) between the Illusion (median [IQR] = 27 [17–31]) and Non-illusion groups (median [IQR] = 20 [15–25]). A post hoc power analysis using G*Power 3.1.9.2 (Faul et al., 2007) indicated that the achieved power was low (Power ≈ 0.307). For the BDI-II, there was no significant difference (*U* = 99.500, *r* = 0.042, *p* = 0.824) between the Illusion (median [IQR] = 8 [5–9]) and Non-illusion groups (median [IQR] = 6 [5–11]). A post hoc power analysis using G*Power 3.1.9.2 (Faul et al., 2007) indicated that the achieved power was low (Power ≈ 0.054). In the HSPS, there was no significant difference (*U* = 120.500, *r* = 0.215, *p* = 0.244) between the Illusion (median [IQR] = 81 [73–92]) and Non-illusion groups (median [IQR] = 92 [78–101]). A post hoc power analysis using G*Power 3.1.9.2 (Faul et al., 2007) indicated that the achieved power was low (Power ≈ 0.44).

## 4 Discussion

The purpose of this study was to investigate whether the illusion of a sense of body lightness can be induced by applying a visual delay system. Our previous study (Hayashida et al., 2025) showed that subjectively preceding feedback can generate the illusion of a sense of body lightness; however, as it was based only on the introspective reports of a very small number of participants, it was uncertain whether the subjectively preceding feedback was actually generated and thereby caused this illusion. All the current participants in the Illusion group reported that a sense of body lightness was induced only when they were given subjectively preceding feedback. Identifying participants who could be classified into the Illusion group largely achieved the primary purpose of this study. However, the proportion was only approximately one-third, and the reasons for this and the underlying mechanisms remain largely unclear. In the following sections, we discuss these issues cautiously and aim to develop this preliminary, exploratory study into future research.

The lower congruence response rate in the Lightness condition than in the Minimum delay condition indicates that incongruence occurred when inducing the illusion of a sense of body lightness. There was no significant difference in the congruence response rate between the Lightness and Heaviness conditions. This might indicate that a sense of bodily lightness, as opposed to a sense of bodily heaviness, is not unique when considering mechanisms of body-sensation illusions based on sensorimotor incongruence. In addition, the results of the two-way ANOVA showed that there was no difference between the groups, and the Heaviness condition was similar to that of the Non-illusion group, indicating that the Illusion group was not uniquely less likely to feel a sense of body heaviness.

In studies using time delay tasks, it is common to fit a logistic curve to the Yes/No response probability for Q1 across delay times and to treat its slope (i.e., sensitivity) and the point of subjective equality (PSE) as the primary dependent variables (Franck et al., 2001; Shimada et al., 2009; Shimada et al., 2010; Kannape and Blanke, 2013; Shimada et al., 2014); however, we chose an analysis procedure based on Q2 for the following reasons. First and foremost, the main purpose of this study was to investigate whether or not there are people who feel a sense of lightness. Therefore, first of all, it was necessary to make groupings. Second, the analysis of congruence using a time delay task generally uses a method that regresses the amplification of the incongruent response rate as the delay level increases on a logistic curve (S-curve). In this case, however, a sine-like curve (rather than an S-curve) is assumed in which the discrepant response rate is amplified for the time being as the delay level increases, but the discrepant response rate decreases owing to the “periodic motion.” Therefore, the sensitivity of congruence cannot be detected by logistic curve regression analysis.

Before considering why incongruency could produce positive effects, we would like to situate the present study methodologically and discuss the future potential of this novel bodily illusion by referring to recent psychophysical literature on bodily illusions. In the present experimental paradigm, the sense of body lightness was assessed using a questionnaire. It is well known that questionnaire-based measures are influenced by introspective ability, cognitive bias, demand characteristics, and memory processes, as such questionnaires are typically administered after illusion induction. To address these limitations, recent work has introduced quantitative, model-based methods to measure the sensitivity of body ownership and embodiment to multisensory integration, including approaches based on signal detection theory and psychometric function estimation (Chancel and Ehrsson, 2020; Lanfranco et al., 2023; Lanfranco et al., 2024; Lanfranco et al., 2025). In these studies, instead of conventional subjective ratings, discriminating body ownership perception in a two-alternative forced-choice task using two rubber hands has been employed. These studies have demonstrated that the multisensory integration window underlying body ownership illusions is considerably narrower—both spatially (Kalckert and Ehrsson, 2014; Lloyd, 2007; Preston, 2013) and temporally (Shimada et al., 2009)—than previously suggested by earlier research. Applying such psychophysical approaches may further enhance the precision with which the body lightness illusion can be elicited and quantified. Although the present report focuses on the presence or absence of the illusion, we believe that this phenomenon holds substantial potential for revealing positive effects in future investigations.

In previous reports, the effects of incongruency were negative sensations, such as a sense of body heaviness or pain. On the other hand, another previous study (Ito et al., 2022) reported the subjectively preceding visual feedback made the perception of muscle fatigue weaker than normal feedback. This previous study and the present study differ substantially in several respects, including the target sensations (perception of muscle fatigue vs. bodily lightness), the context (motor control vs. bodily illusion), and the dynamism of movement (upper-limb movements vs. whole-body walking). In addition, whereas the previous study focused on the alleviation of negative sensations, the present study addresses the generation of a novel bodily illusion. Nevertheless, both studies suggest that the perception of bodily states can be positively modulated by the sign of prediction error, and our findings are therefore conceptually consistent with this framework. That is, the current study may have induced a sense of body lightness because the predicted sensation exceeded the actual sensation. Visual information from the subjectively preceding feedback was given as indicating the current state (actual sensory experience) and may have induced a sense of body lightness by exceeding the predicted body position of the self, based on somatosensory information. This positive effect of the illusion of a sense of body lightness occurred because of the subjectively preceding feedback that could be generated by the combination of the most important features of walking, namely periodicity and the visual delay system. In this sense, our results do not contradict previous reports but rather complement and extend them by showing that sensorimotor incongruence, depending on its direction, can generate not only negative but also positive subjective bodily experiences.

When given positive or negative feedback, whether the feedback is attributed to the self may also be important. In intentional binding experiments, several reports found that the sense of agency increased when the feedback was positive and decreased when feedback was negative (Takahata et al., 2012; Yoshie and Haggard, 2013). From these findings, we can infer that even with a slight incongruence of the subjectively preceding feedback, the cognitive process of attributing positive images to the self may have been executed. The timing of the self-response Q1 and Q2 after the visual feedback in our experiment was optional; thus, it is possible that the participants responded after the cognitive process of attributions or not to themselves. Although the size–weight illusion is conceptualized at the sensorimotor level and relates to vision and proprioception, studies on intentional binding have addressed it at the action level by manipulating reward (Takahata et al., 2012) and emotion (Yoshie and Haggard, 2013). Whether considering the size–weight illusion or intentional binding, although they involve different sensory modalities or computational mechanisms, they share a common feature: positive illusion effects induced by incongruences between prediction and feedback. This suggests that an integrated mechanism encompassing both the sensorimotor level and the action level operates in the background.

These attribution processes should be involved in inducing not only a sense of agency but also a sense of ownership. A previous study successfully induced a sense of ownership and agency in participants toward the avatar’s body, despite the lack of a perfect match between intent and the actual movement (Burin et al., 2020). This indicates a certain degree of flexibility in embodiment sensations. Our procedures may also induce a sense of ownership and agency for the body projected on the screen. For our participants, the “tolerable incongruence” that can induce a sense of ownership may have induced a different sensation, including that of lightness. This previous study compared the first-person perspective with a third-person perspective, and our study corresponds to the third-person perspective. Another difference from the previous study was that in our setup the body shown was not an avatar but the participant’s own projected body, and both the intended movement and the actual movement were those of the participant. Perhaps the sense of ownership could have been enhanced if our procedure had used a first-person perspective, and the effects on congruence response rate and the sense of body lightness might also have been strengthened.

One of the well-known first-person perceptual illusions is vection, the illusory perception of self-motion induced by visual motion. The term vection is defined as the illusory sense of self-motion (Palmisano et al., 2015). A classic example is the train illusion, whereby observing a forward train begin to move creates the illusory sensation that one’s own train, although stationary, is in motion. Although vection can be induced and modulated through various senses, including auditory (Väljamäe, 2009), tactile (Kooijman et al., 2022) and biomechanical stimulation (Ash et al., 2013), this phenomenon can mainly occur when misleading visual signals convey a sense of motion, overriding the vestibular signals that would otherwise indicate a lack of movement. It has been suggested that, under conditions in which vestibular signals are weak, the brain may generate an illusion by interpreting wide-field visual motion as self-motion (McManus and Harris, 2023). If the present experimental paradigm had been conducted from a first-person perspective, the sensory modality modulated by the visual stimulus might have been the vestibular sense, potentially inducing a sense of body lightness originating from vestibular signals. In contrast, in the present study, the illusion appears to have arisen from mismatches in the positions of the upper and lower limbs. That is, in the current experimental paradigm, the sensory modality modulated by the visual stimulus may have been proprioception, and the induced sense of body lightness may therefore have originated from proprioceptive signals. An integrated understanding of sense of body lightness—namely, whether it arises from a specific sensory modality or from the combined contribution of multiple modalities—would be desirable.

There were no significant differences between groups on any of the psychological measures assessed in this study. Although the mechanisms underlying the sense of agency and the sense of bodily lightness partly overlap in that both involve monitoring the congruence between sensory prediction and actual sensory feedback, the present results suggest that the psychological constructs measured in this study are not shared. In a previous study, the cutoffs for the SPQ were 41 and 12 (Raine, 1991). The means for both groups were within the cutoffs. The BDI-II classifies the lowest severity of depression at 13 or below (Beck et al., 1996). The participants in this study were within this range. As depression is associated with a sense of agency (Obhi et al., 2013; Malik and Obhi, 2019; Nobusako et al., 2020), if BDI-II values were dispersed, they might have also affected the sense of agency and sense of body lightness Although there is no clear cutoff for the HSPS, the mean score of 86.68 ± 12.70 from Takahashi (2016) is similar to that of both groups and seems to be an average value. Similar to depression, this value could be related to the sense of agency if sufficient variance were present.

This study has several limitations that should be resolved. First, individual differences in stride times were not considered. To generate the subjectively preceding feedback, a delay time close to that of one stride was required. For example, the meaning of a delay time of 1,000 ms would differ for participants with a single stride time of either 1,000 ms or 1,200 ms; if a participant with a stride of 1,000 ms was given feedback with a delay of 1,000 ms, he or she would almost feel this as being real-time feedback. Hence, the insertion of a 1,200 ms delay would be necessary for a participant with a stride time of 1,200 ms to feel this as being real-time feedback. As the 200 ms delay in our study (Hayashida et al., 2025) was noticed by most participants, if the setting was 200 ms shorter than the individual’s stride time (if the single stride time was 1,000 ms, the delay time would be 800 ms), some participants in the Non-illusion group might have experienced the subjectively preceding feedback.

To address this issue, robustness could be improved by measuring each participant’s stride time in advance and setting individualized delay durations based on that value. In addition, the question of how much temporal mismatch is tolerable remains unresolved; therefore, it would be necessary to utilize mismatch detection sensitivity assessed by Q1. For example, if a participant’s 50% threshold for mismatch detection is 200 ms, it would be necessary to provide predicted feedback that exceeds 200 ms by a small margin.

Second, the temporally incongruent visual feedback used in this study was from a third-person perspective. This is based on our previous study (Hayashida et al., 2025). However, the visual feedback originally provided was from a first-person perspective. For example, temporal incongruence between first-person perspective visual feedback, such as optical flow (Warren et al., 2001; Fajen, 2013), and walking speed may induce a strong sense of body lightness or heaviness. As the first-person perspective involves visual feedback with which we are familiar, it may be able to induce a sense of body lightness better than a third-person perspective can. This issue can be solved immediately with the use of virtual reality technology. Third, it is unclear whether it is possible to counteract this and create the illusion of body lightness in patients who complain of a sense of body heaviness. Patients may have incongruities in many aspects of motor, sensory, and cognitive functions. As the manipulations used in the current study were based on visual information only, future studies should clarify whether incongruence can be corrected across sensory modalities. Fourth, we believed that this experimental paradigm could be related to the sense of body ownership. As the participants knew that what they saw on the screen was themselves, the integration of visual and somatosensory perception might have induced a sense of ownership. To investigate the extent to which this sense of body ownership is involved in inducing the sense of body lightness, it is necessary to quantify the sense of body ownership on a Likert scale in future studies. In our experiment, we can only indicate that it is a phenomenon based on the relative timing of tactile, proprioceptive, and visual feedback.

The most important characteristic of walking, periodicity, was involved in inducing the illusion of a sense of body lightness through the interesting phenomenon of subjectively preceding feedback. We propose that this phenomenon is an important discovery that could not have been found in previous experiments using upper-extremity tasks only. This new kind of illusion could be adapted to physical movements other than walking and developed into an interdisciplinary study. Although its adaptations, limitations, and mechanisms remain unknown, we believe that, in the near future, the development of this study may help patients suffering from unpleasant subjective experiences, such as a sense of body heaviness or pain associated with movement caused by incongruence.

Future studies should be designed with adequate *a priori* statistical power to detect small-to-moderate group differences in psychological scale scores. Based on the observed effect sizes in the present study, larger sample sizes will be required to reliably identify between-group differences using nonparametric tests. Importantly, a priori sample size planning informed by theoretically or clinically meaningful effect sizes, rather than *post hoc* estimates, will be essential to improve the robustness and interpretability of future findings.

## 5 Conclusion

Herein, we report an experience of the illusion of a sense of bodily lightness while walking. This illusion, related to the mechanism of sensorimotor incongruence, was induced by subjectively preceding feedback through the application of a visual delay system during walking. This study was preliminary and exploratory in nature and included several limitations and unresolved issues. However, this new and simple idea may help individuals who suffer from unpleasant subjective experiences caused by incongruence in the future.

## Data Availability

The original contributions presented in the study are included in the article/Supplementary material, further inquiries can be directed to the corresponding author.
